# Plasticity of patient-matched normal mammary epithelial cells is dependent on autologous adipose-derived stem cells

**DOI:** 10.1038/s41598-019-47224-2

**Published:** 2019-07-24

**Authors:** Annika Kengelbach-Weigand, Kereshmeh Tasbihi, Pamela L. Strissel, Rafael Schmid, Jasmin Monteiro Marques, Justus P. Beier, Matthias W. Beckmann, Reiner Strick, Raymund E. Horch, Anja M. Boos

**Affiliations:** 1 Department of Plastic and Hand Surgery and Laboratory for Tissue Engineering and Regenerative Medicine, University Hospital of Erlangen, Friedrich-Alexander University of Erlangen-Nürnberg (FAU), Erlangen, Germany; 2 Department of Obstetrics and Gynecology, University Hospital of Erlangen, Laboratory for Molecular Medicine and Comprehensive Cancer Center Erlangen-EMN (CCC), Friedrich-Alexander University of Erlangen-Nürnberg (FAU), Erlangen, Germany; 3 Present Address: Department of Plastic Surgery, Hand and Burn Surgery, University Hospital RWTH Aachen, Aachen, Germany

**Keywords:** Cell growth, Cancer, Cell biology

## Abstract

Due to the increasing clinical application of adipose-derived stem cells (ADSC), e.g. lipotransfer for breast reconstruction, this study aimed to gain novel insights regarding ADSC influence on breast tissue remodeling and determine patient-dependent factors affecting lipotransfer as well as begin to address its oncological risks. The ADSC secretome was analyzed from five normal breast reduction patients and contained elevated levels of growth factors, cytokines and proteins mediating invasion. ADSC/ADSC secretomes were tested for their influence on the function of primary mammary epithelial cells, and tumor epithelial cells using cell culture assays. ADSC/ADSC secretomes significantly stimulated proliferation, transmigration and 3D-invasion of primary normal and tumor epithelial cells. IL-6 significantly induced an EMT and invasion. The ADSC secretome significantly upregulated normal epithelial cell gene expression including MMPs and ECM receptors. Our study supports that ADSC and its secretome promote favorable conditions for normal breast tissue remodeling by changing the microenvironment. and may also be important regarding residual breast cancer cells following surgery.

## Introduction

Within recent years, the application of adipose-derived stem cells (ADSC) for Reconstructive and Plastic Surgery has tremendously increased. ADSC are mesenchymal stem cells (MSC) that are known for their multilineage differentiation potential, including lipocytes and osteocytes^1^, cardiomyocytes^2^, endothelial cells^3^, myoblasts^4^ and possibly even mammary gland-like epithelial cells^5,6^. They not only have the capability to replace damaged tissue by differentiating into other phenotypes, but they are also known for their secretion of various proteins, cytokines and growth factors that can stimulate local cells to migrate, proliferate and differentiate into a damaged area^7^.

As part of the lipotransfer procedure, e.g. for breast reconstruction after breast cancer surgery, ADSC are increasingly in the focus of clinicians and basic scientists due to their potential effects and possible side effects. Fat transplantation has been published as early as 1893, when Neuber first transplanted autologous fat from the upper arm to treat a facial contour defect^8^. Two years later, the German surgeon Czerny performed the first lipotransfer using a lipoma for reconstruction of the breast^9^. Today, lipotransfer is mainly performed as an adjunct to other established reconstructive procedures.

At transplantation sites autologous tissue can be easily harvested and molded. Side effects of alloplastic implants including rupture, capsule contraction or development of BIA-ALCL (breast implant-associated anaplastic large cell lymphoma)^10^ due to synthetic materials, can be avoided with the use of natural tissues^11^. However, a major drawback of fat grafting is the unpredictability of the clinical outcome since high volume absorption rates are common. An average of 50% volume loss should be taken into account not only due to hypoxia, but also due to later remodeling processes even after several months to years after reconstruction^12^. To improve fat graft survival, the cell-assisted lipotransfer (CAL) was first described eleven years ago by Matsumoto *et al*. as a promising novel technique^13^. ADSC are concentrated during the CAL to support neoangiogenesis and survival of the injected lipoaspirate^13^. However, data about the efficacy and safety of CAL compared to conventional lipotransfer are not consistent. While some studies confirm a higher tissue survival rate of CAL for breast reconstruction, others could not find any statistical difference and its efficacy remains debatable (reviewed in^14,15^).

Since CAL has entered into more routine clinical practice, especially for breast cancer patients, there are concerns about a detrimental effect on the recurrence rate. Notably, preclinical data show stimulation of breast cancer cells by ADSC^16–18^. In contrast, some clinical studies could not confirm this effect resulting in no higher recurrence rate of breast cancer after lipotransfer^19–21^. These conflicting data are currently in the focus of scientific discussion. Additional problems among clinical studies are inadequate control groups, short follow-up periods, small patient groups, different surgical techniques and the frequent use of immortalized cell lines^22^.

Unraveling the biology of normal breast tissues is of utmost importance to understand the cellular dynamics following lipotransfer for breast reconstruction, including possible effects on breast cancer patients. This present experimental study tested if ADSC and their secreted factors, defined as the ADSC secretome, could exert a stimulatory or inhibitory effect on patient-matched primary mammary epithelial cells (MEC) using a variety of functional cell culture analyses. Our main goal was to gain a better understanding of normal breast cell interaction which could relate to 1) clinical results of efficacy of lipotransfer and 2) evaluate possible patient-dependent factors associated with lipotransfer. Additionally, we initiated preliminary studies to begin to address oncological safety of liptransfer. In order to mimic the clinical scenario and in contrast to other investigations implementing purchased cell lines; we used primary patient-derived cells in early passages isolated according to our previous study^23^. We propose that this current investigation will help to contribute to a better understanding of cell-cell interactions within breast tissue with regards to lipotransfer and possibly breast cancer.

## Materials and Methods

### Patients

Clinical data from five healthy females (NORMA1-5, age 41.2 ± 14.6 yr, BMI 29.6 ± 4.3 kg/m²) with medically indicated breast reduction surgeries and from one patient with an invasive inflammatory ductal breast carcinoma (IFDUC1) (age 46 yr) are described in Table 1.

**Table 1:** Patient data.

Patient	Age	BMI (kg/m²)	Smoking
NORMA1 (MEC + ADSC)	32	27.68	no
NORMA2 (MEC + ADSC)	22	22.95	yes
NORMA3 (MEC + ADSC)	43	31.23	no
NORMA4 (MEC + ADSC)	49	32.60	no
NORMA5 (MEC + ADSC)	60	33.46	no
IFDUC1 (MEC)	46	n. a.	n. a.

ADSC, adipose-derived stem cells; IFDUC, invasive inflammatory ductal breast carcinoma; MEC, mammary epithelial cells; NORMA, normal mammary cells.

Human tissue collection was approved by the Ethics Committee of the University of Erlangen-Nürnberg (Germany) (Ethics #264_13B, #99_15 Bc) in accordance with the World Medical Association Declaration of Helsinki. Informed consent was obtained from all patients^23^.

### Cultivation of normal and tumor mammary epithelial cells (NORMA and IFDUC MEC) and normal adipose-derived stem cells (ADSC)

Previously derived primary cell lines representing normal mammary epithelial cells (NORMA1-5 MEC), and patient-matched normal adipose-derived stem cells (ADSC1-5) and IFDUC1 MEC were analyzed in this present study^23,24^.

For cell culture studies NORMA MEC and IFDUC MEC were cultivated in 75-cm^2^ culture flasks in Mammary Epithelial Cell Growth Medium (MECGM) (Provitro GmbH, Berlin, Germany), ADSC in MEM alpha medium (Life technologies, Carlsbad, California, USA) supplemented with 10% fetal calf serum (FCS) (FBS superior, Biochrom) at 37 °C and 5% CO_2_. Medium was changed every 2–3 days. Experiments were done with cells in passage 5–7. Testing of mycoplasma contamination was performed regularly (AppliChem GmbH, Darmstadt, Germany).

### Preparation of ADSC conditioned medium (CM) or the ADSC secretome

ADSC were seeded into 75-cm^2^ culture flasks in MEM alpha with 10% FCS and cultivated at 37 °C, 5% CO_2_ until 90% confluency. The medium was discarded and the cells washed with PBS. ADSC were then cultivated in newly added 10 ml MEM alpha without FCS for 24 h. CM (ADSC secretome) was concentrated using filter tubes (Amicon^®^ Ultra-15 Centrifugal Filter Devices, 3 K; Merck KGaA, Darmstadt, Germany) at 4,000 g for 30 min. The ADSC secretome was used in 3-fold concentration, where the ultrafiltrate from 10 ml was diluted with Mammary Epithelial Cell Basal Medium (MECBM) containing 5% of the supplement mix of the amount that is normally used in the MECGM (in the following described as “reduced MECGM”). Control Medium was incubated for 24 h at 37 °C and 5% CO_2_ and concentrated as above.

### Analysis of the ADSC secretome

For detection of the relative expression of 1,000 different proteins, the Human L-series 1,000 Antibody Array Glass (RayBiotech Inc., Norcross, Georgia, USA) was used. ADSC secretomes from all patients were pooled and the assay was performed and evaluated by the service of tebu-bio (Le-Perray-en-Yvelines, France).

For further analyses, an array-based multiplex ELISA (Quantibody^®^, RayBiotech Inc./tebu-bio) for chemokine (C-C motif) ligand 5 (CCL5), tumor necrosis factor-beta (TNF-β), hepatocyte growth factor (HGF), stromal cell-derived factor 1 (SDF1)/also known as C-X-C motif chemokine 12 (CXCL12), monocyte chemotactic protein 1 (MCP1)/also known as chemokine (C-C motif) ligand 2 (CCL2), transforming growth factor beta 1 (TGF-β1), C-X-C motif chemokine ligand 1 (CXCL1), basic fibroblast growth factor (bFGF), Dickkopf homolog-1 (DKK1), thrombospondin-1 (THBS1), matrix metalloproteinase-1 (MMP-1) was performed. Single ELISAs for fibronectin (FN) (RayBiotech Inc.), ectonucleotide pyrophosphatase/phosphodiesterase 2 (ENPP2)/also known as autotaxin (ATX), interferon gamma (IFNγ), TNF-α, interleukin 6 (IL-6) and IL-8 were performed.

### Cell proliferation and viability

2,000 NORMA MEC or 5,000 IFDUC1 MEC were seeded in triplicates into three 96-well plates in MECGM and incubated at 37 °C and 5% CO_2_ for 24 h. The medium was removed and replaced by 100 µl 3-fold concentrated patient-matched ADSC secretomes for NORMA MEC and for IFDUC1 MEC the ADSC4 secretome. For NORMA MEC the assay was performed 3x and for IFDUC1 MEC 2x, where three technical replicates were used for statistical analyses. Cell viability was measured using the Colorimetric Cell Viability Kit I (WST-8) according to the manufacturer’s instructions (PromoCell GmbH, Heidelberg, Germany) after an incubation period of 24 h, 48 h and 72 h (Multiskan^TM^ GO, Thermo Fisher Scientific, Waltham, MA, USA). Briefly, 10 µl of CCVK-I solution were added to each well and incubated for 2 hours at 37 °C. Absorbance was measured at 450 nm, background at 600 nm was subtracted.

### Migration assay

For the migration assay 100,000 NORMA MEC were seeded in duplicates in a 24-well plate in MECGM and incubated for 24 h at 37 °C and 5% CO_2_. A scratch was generated by using a 100 µl pipette tip and the cells were washed and then incubated with 500 µl of a 3-fold concentrated ADSC secretome or control medium. At 0 h, 6 h, 12 h and 24 h 4 images at defined regions along the scratch were taken in 40-fold magnification (Olympus IX83, cellSens Software). Migration of the cells was analyzed by measurement of the uncovered area with the TScratch software developed by the Koumoutsakos group (CSE Lab), at ETH Zurich^25^. The assay was performed three times.

### Invasion and transmigration assay

For the invasion and transmigration, Boyden chambers of Corning^®^ Transwell^®^ polycarbonate membrane inserts (Corning Inc., Corning, New York, USA) were used. For the invasion assay, transwells were coated with collagen at a concentration of 2.4 mg/ml (Collagen Type I solution from bovine skin; Sigma-Aldrich, St. Louis, Missouri, USA). Transwells were transferred into a 24-well plate and seeded with 50,000 NORMA MEC in duplicates with reduced MECGM and the assay performed 3x. For IFDUC MEC1 30,000 cells were seeded per well in triplicates for the invasion assay and duplicates for transmigration. The assay was performed 1x where technical triplicates for the invasion assay were used for statistical analyses. The lower chamber was filled with either a 3-fold concentrated ADSC secretome or a 3-fold concentrated control medium. After 8 h at 37 °C and 5% CO_2_, the transwell chambers were washed, fixed with ice-cold methanol and stained with DAPI (1 µg/ml, 10 min) (Life technologies, Carlsbad, California, USA). The cells in the inner part of the transwells were wiped with a PBS-coated cotton swab in twisting motion to remove the non-migrated and non-invaded cells, respectively. Cells that migrated through the membrane were counted in four regions of interest (ROI) per transwell (one image per quadrant) in 200-fold magnification (Olympus IX83, cellSens Software, Olympus Corporation, Tokio, Japan).

### 3-D collagen invasion assays with IL-6 and IL-8

Cell invasion assays were performed and analyzed according to^23^. Collagen beds in 12-well plates for invasion were prepared as previously described^23^.

For cell culture set-up, 30,000 cells of NORMA1 MEC, NORMA2 MEC, NORMA4 MEC and IFDUC1 MEC, were added to the collagen beds in triplicates in reduced MECGM with different supplements. NORMA MEC and IFDUC1 were incubated either with patient-matched ADSC secretomes or the ADSC4 secretome, respectively, control medium, or incubated with 100 ng/ml IL-6 or 100 ng/ml IL8 or a control medium without cytokines for the particular cell type. After an incubation time of 72 h, cells were fixed in 4% paraformaldehyde. All invaded cells were counted in >20 optical fields (Carl Zeiss AG, Oberkochen, Germany) per well and represented as number of invaded cells per cm^2^ according to^26,27^.

### Proliferation in direct co-culture

For evaluation of proliferation in a direct co-culture system, we included three different direct co-culture conditions: MEC were cultivated with 1) ADSC, 2) dermal fibroblasts, or 3) as MEC monocultures. First, 8,000 ADSC were seeded in MEM alpha containing 10% FCS into 48-well plates. Second, a mesenchymal control of 8,000 human dermal fibroblasts (Provitro GmbH) was seeded in DMEM (Life technologies) containing 10% FCS. Third, as a second test group 8,000 NORMA MEC were seeded in MECGM. All cells were seeded in duplicates. After 24 h at 37 °C and 5% CO_2_, cell layers were washed and then 8,000 NORMA MEC were labeled with CellTracker™ CM-DiI Dye (Life technologies) and added to each well in reduced MECGM. Cell labeling was performed using 5 µg/ml CM-DiI Dye, incubation at 37 °C for 5 min and further incubation at 4 °C for 15 min. After 3 d, cells were fixed, permeabilized and after blocking with 5% goat serum for 30 min, incubated with a Ki67 antibody (0.46 µg/ml, monoclonal mouse anti-human Ki67 antigen clone MIB-1; Dako, Glostrup, Denmark) at room temperature (RT). For visualization, the secondary antibody Alexa Fluor^®^ 488 goat anti-mouse IgG1 Antibody (10 µg/ml; Life technologies) was used for 30 min at RT. Controls were performed using isotype (mouse IgG1, negative control; Dako), primary and secondary antibody controls. For quantification of the proliferation of NORMA MEC, double-labeled (Ki67 and CM-DiI Dye) cells were counted in 200-fold magnification in four ROIs and analyzed in relation to each other (Olympus IX83, cellSens Software). The assay was performed 3x.

For microscopic evaluation 30,000 ADSC or human dermal fibroblasts were seeded into 6-well plates in MEM alpha and DMEM, respectively, containing 10% FCS. After 24 h cells were labeled with 10 µM CellTrace™ Oregon Green^®^ 488 Carboxylic Acid Diacetate, Succinimidyl Ester (Life technologies) for 15 min at 37 °C. CM-DiI-Dye-labeled NORMA MEC were added in MECGM. After 24 h the medium was changed to reduced MECGM. Pictures were taken after 3 d. Life cell imaging was performed for 24 h at 40-fold magnification (Olympus IX83, cellVivo, cellSens Software).

### Indirect co-culture

For indirect co-culture experiments, a 6-well transwell system was used with MEC seeded in the bottom and ADSC in the upper part of the chambers. 50,000 NORMA MEC were seeded in duplicates in MECGM. After 24 h at 37 °C and 5% CO_2_ the medium was replaced by 3.5 ml reduced MECGM. 100,000 ADSC were then seeded in the transwells (6 Well ThinCert™ Cell Culture Inserts, Greiner Bio-One, Frickenhausen, Germany) in reduced MECGM. Instead of ADSC, in the first control group a mesenchymal cell control, 100,000 human dermal fibroblasts were used and in the second control group, 100,000 NORMA MEC were used. Images were taken after 3 d (Olympus IX83, cellVivo, cellSens Software) and RNA isolation was performed. The assay was performed three times.

For membrane staining, the same experimental setting was used in 48-wells. After 3 d of incubation, cells were fixated with 4% paraformaldehyde for 15 min at RT. Wheat Germ Agglutinin, Tetramethylrhodamine Conjugate (10 µg/ml; Life technologies) was incubated for 10 min at RT. Cell nuclei were stained with DAPI (1 µg/ml, 10 min).

### RNA isolation, cDNA synthesis and real-time PCR

RNA of all probes was extracted using the RNeasy Mini Kit with the corresponding QIAshredder Homogenizer (Qiagen, Hilden, Germany). RNA was reverse-transcribed into cDNA with QuantiTect Reverse Transcription Kit with a DNase I incubation (Qiagen). For quantitative real-time PCR, “SsoAdvanced Universal SYBR Green Supermix” (Bio-Rad Laboratories, Hercules, California, USA) was used with a Light Cycler (Bio-Rad CFX96). All kits were used according to the manufacturers’ protocols. Samples were tested as triplicates. tyrosine 3-monooxygenase/tryptophan 5-monooxygenase activation protein, zeta (*YWHAZ*) was used as a housekeeping gene. Data analysis was performed using the 2^−ΔΔCT^ or 2^−ΔCT^ method. Table 2 specifies all primers used for real-time PCR.

**Table 2:** Primer sequences.

gene	Forward 5′-3′	Reverse 5′-3′
*ACTA2*	ACTGCCTTGGTGTGTGACAA	CGTCCCACAATGGATGGGAA
*CCNB1*	CTGCTGGGTGTAGGTCCTTG	TGCCATGTTGATCTTCGCCT
*CD105*	CCAAGACCGGGTCTCAAGAC	TGTACCAGAGTGCAGCAGTG
*CD24*	CCAGTGAAACAACAACTGGAAC	TGCAGAAGAGAGAGTGAGACCA
*CD29*	GTCGTGTGTGTGAGTGCAAC	TTGCAGATCTGTCCGTTGCT
*CD44*	CTCCAGTGAAAGGAGCAGCA	GCAGGGATTCTGTCTGTGCT
*CD73*	TATCCGGTCGCCCATTGATG	ACGCTATGCTCAAAGGCCTT
*CD90*	ATCAGGAGTTCCAGTGCTGC	TGGCTTCCCTCTTCACGAAC
*CDH1*	TCATGAGTGTCCCCCGGTAT	TCTTGAAGCGATTGCCCCAT
*CNN1*	TTGAGGCCAACGACCTGTTT	TTTCCGCTCCTGCTTCTCTG
*COLA4A1*	CAGGTCCTTACGACATCATCAA	ACCAATCCTGTAACACCTTGCT
*CTSV*	ACGTGACGCCAGTGAAGAAT	GCTCGCTCAGTGAGACAAGT
*FN1*	GAGAAGTATGTGCATGGTGTCAG	AATACTTCGACAGGACCACTTGA
*BIRC5*	TCTGTCACGTTCTCCACACG	CAGTAGGGTCCACAGCAGTG
*ITGA5*	AGAAGACAACATCTGTGTGCCT	AAGTGAGGTTCAGGGCATTCTT
*ITGA6*	AGGATGGGTGGCAAGATATAGTT	CATTAAGACGAATTGGCTTCACATT
*KRT 14*	CAGTCCCAGCTCAGCATGAA	GCATGCAGTAGCGACCTTTG
*KRT 18*	ACAAGTACTGGTCTCAGCAGATTG	CTCCAAGGACTGGACTGTACG
*KRT 19*	AGGAGGAAATCAGTACGCTGAG	ATATTGGCTTCGCATGTCACT
*KRT 5*	TGTTCGAGCAGTACATCAACAAC	ATGTTTCTCAGCTCTGAGTCCAG
*KRT 7*	ACAGCTGCTGAGAATGAGTTTGT	GGAAGTTGATCTCATCATTCAGG
*KRT 8*	GGAAGCTGGTGTCTGAGTCC	CTGTTCCCAGTGCTACCCTG
*MME/CD10*	GATAAGTGGAGCAGCTGTAGTCAA	CATAGTTCAATGAGTTGGACTGCT
*MMP2*	GCCGTGTTTGCCATCTGTTT	AGCAGACACCATCACCTGTG
*MMP3*	TGAACCTGAATTGCATTTGATCTC	CGAGGTCCTTGCTAGTAACTTCA
*MMP7*	GAGATGCTCACTTCGATGAGG	GGATCAGAGGAATGTCCCATAC
*MMP14*	CAACACTGCCTACGAGAGGAA	CTCCTTAATGTGCTTGGGGTAG
*SNAI1*	GCCCCACAGGACTTTGATGA	CCCTCCACAGAAATGGCCAT
*TP53*	GAAAACCTACCAGGGCAGCT	GGGAGTACGTGCAAGTCACA
*VIM*	AATCCAAGTTTGCTGACCTCTC	GTCTCCGGTACTCAGTGGACTC
*YWHAZ*	ATGAGCTGGTTCAGAAGGCC	AAGATGACCTACGGGCTCCT

### Statistics

Data are expressed as the mean ± standard error of the mean or the mean ± standard deviation. Statistical analysis was performed using SPSS 20.0 for Windows (SPSS Inc., IL, USA). Results were interpreted by Mann-Whitney U Test due to the small sample size. The level of statistical significance was set to p ≤ 0.05. A p-value ≤ 0.01 was considered to be highly significant. The graphics were created with Origin 8.5.1 G and CorelDRAW X5.

## Results

### Patient-matched NORMA ADSC and MEC and IFDUC1 MEC

In our previous investigation we established primary cell lines and characterized NORMA MEC and ADSC from five different patients and one IFDUC using gene and protein expression and differentiation assays^23^. For example, ADSC showed a mesenchymal phenotype with differential expression of typical stem cell markers and NORMA MEC expressed breast cell luminal cytokeratin (*KRT) 7, 8, 18*, *19* and basal *KRT 14, 5* markers indicating the presence of both epithelial phenotypes^23^. IFDUC1 MEC was clonal and represented more of a basal-like epithelial cell lineage expressing differential levels of *epithelial cell adhesion molecule* (*EPCAM), CD49f, MUC1, KRT8/1814, CD44* and *CD24*^23^. Furthermore, there was support for bi-potent undifferentiated IFDUC1 MEC cells (*CD10, CD34*+, *CD45*+).

### The ADSC secretome

**Figure 1:**
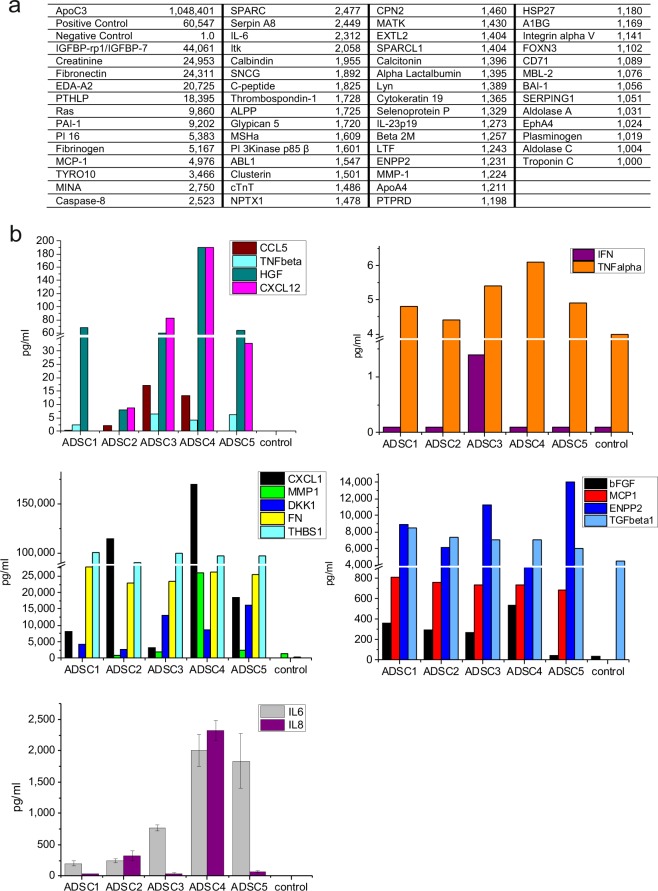
Analysis of the ADSC secretome. A profiling of pooled ADSC CM was performed by using an antibody array of 1,000 proteins. Negative control (control medium) was set to 1.0. The relative protein expression in ADSC CM is indicated as x-fold value compared to control medium (**a**). With an array-based multiplex ELISA the concentrations of CCL5, TNF-β, HGF, CXCL12, IFNγ, TNF-α, CXCL1, MMP1, DKK1, FN, THBS1, bFGF, MCP1, ENPP2, TGF- β1, IL-6 and IL-8 were quantified (pg/ml) (**b**).

In order to design experiments to test the influence of the ADSC secretome on NORMA MEC and IFDUC1 functions, we profiled pooled ADSC1-5 CM using an antibody array identifying 1,000 proteins. Compared to a negative control (set to 1.0), a range of proteins was identified in high amounts in ADSC secretome samples which showed relative signal intensities over 1,000 compared to the negative control (e. g. IGFBPs: 44,061; IL-6: 2,312; MMP-1: 1,224; FN: 24,311; THBS1: 1,728; MCP-1: 4,976 and ENPP2: 1,231) (Fig. 1a). We also used an array-based multiplex ELISA to identify additional proteins in non-pooled patient specific ADSC CMs; (CCL5, TNF-β, TNF-α, HGF, IFNγ, CXCL12, DKK-1, bFGF, TGF-β1, IL-6 and CXCL1) (Fig. 1b).

Secretion of most growth factors differed among the patients. For example, highest amounts of HGF, CXCL12, CXCL1, and MMP1 were detected in the ADSC4 secretome. In contrast, the ADSC1 secretome showed no detectable levels of CXCL12 or MMP-1. However, amounts of FN, THBS1, MCP1, ENPP2 and TGF-β1 were in a similar range. In all patients, the concentration of TNF-β, TNF-alpha were lower in the range of 5 pg/ml but Interferon gamma (IFNγ) was virtually non-detectable. IL-6 and IL-8 were measured in every patient ADSC secretome and showed differences. For example, in ADSC1 and ADSC2 the IL-6 concentration was low (0.2–0.24 ng/ml), however, ADSC4 and ADSC5 showed high levels (1.8–2.0 ng/ml). For ADSC4 the IL-6 and IL-8 (2.3 ng/ml) was approximately 10–60-fold higher when compared to the other patients. Since ADSC secretomes support a possible regulation of cell growth (e.g. IGFBPs, HGF), migration and invasion (e.g. TGFβ, TNFα/β, FN, MMP, IL-6, IL-8) we performed experiments below implementing NORMA and IFDUC MEC cells.

### The ADSC secretome stimulates cell viability/proliferation and migration of NORMA and IFDUC1 MEC

**Figure 2:**
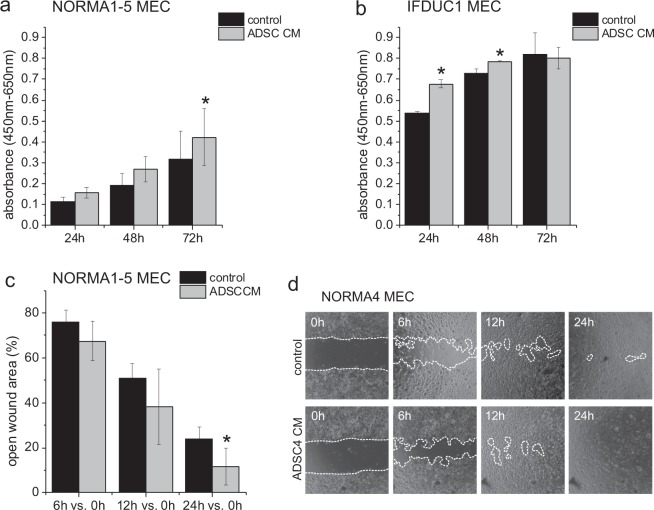
WST-8 assay of NORMA1-5 and IFDUC1 MEC and migration assay of NORMA1-5 MEC cultivated in ADSC secretome. Bar graphs show the viability stimulation represented in absorbance (y-axis) ± SD of NORMA1-5 MEC (x-axis) cell cultures in the presence or absence of patient-matched ADSC secretome at 24–72 h (**a**). Bar graphs show the viability stimulation represented in absorbance (y-axis) ± SD of IFDUC1 MEC (x-axis) in the presence or absence of ADSC4 secretome at 24–72 h (**b**). Bar graphs show the of open wound area represented in percentage (y-axis) ± SD compared to 0 h of NORMA MEC (x-axis) cell cultures in presence or absence of ADSC secretome (**c**). Representative pictures of NORMA4 MEC migration in control and ADSC4 secretome (**d**). (*p ≤ 0.05).

When NORMA1-5 MEC were cultivated in the presence of patient-matched ADSC secretomes, cell viability and proliferation kinetically increased from 24–72 hours where 72 h (about 2.5-fold) was significant compared to controls (Fig. 2a). Similarly, the ADSC secretome significantly stimulated cell viability and proliferation of IFDUC1 MEC after 24 h and 48 h (Fig. 2b). In addition, when NORMA1-5 MEC were cultured with ADSC secretomes a kinetic increase of migration was observed with a significant difference by 24 h compared to controls (Fig. 2c/d).

### Transmigration and invasion of NORMA and IFDUC1 MEC are significantly increased by the ADSC secretome

**Figure 3:**
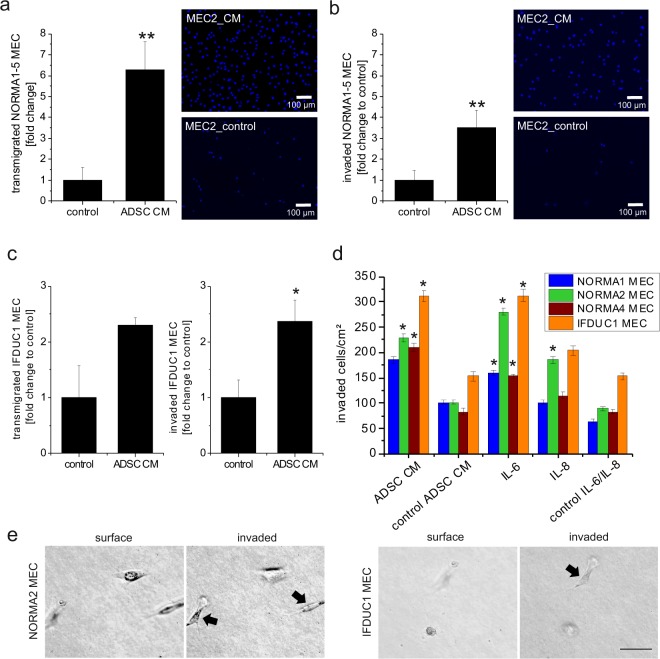
Transmigration and invasion of NORMA MEC and IFDUC1 MEC in ADSC secretome and IL-6/IL-8. ADSC secretome significantly stimulated transmigratory and invasive properties of NORMA1-5 MEC (**a/b**) and IFDUC1 MEC (**c**). Bar graphs show fold change of the number of transmigrated cells (y-axis) or invaded cells in collagen (y-axis) ± SD of NORMA1-5 MEC (**a/b**) or IFDUC1 MEC (**c**) (x-axis) compared to control in the presence of patient-matched ADSC secretome or the ADSC4 secretome at 24–72 h, respectively. Example pictures showing Boyden chamber results of migrated or invaded NORMA2 MEC (stained with DAPI) incubated with and without ADSC secretome (**a/b**). Bar graphs show a comparison of the number of invaded cells through collagen (y-axis) of NORMA1-4 MEC and tumor IFDUC1 (x-axis) cell cultures ± SEM in the presence or absence of patient-matched ADSC secretome or IL-6 and IL-8 at 3 days (**d**). On the surface of collagen beds, MEC had the typical round-shaped morphology. EMT led to invasion of both NORMA and IFDUC1 MEC (bar = 50 µm) (**e**). (*p ≤ 0.05; **p ≤ 0.01).

We next tested the ADSC secretome influence on migration and 3-D invasion using transwells. Results showed that cellular transmigration of NORMA1-5 MEC was highly significantly increased in the presence of patient-matched ADSC sectretomes compared to controls (Fig. 3a). Results of 3-D invasion assays showed a highly significant induction of invading cells in the presence of ADSC sectretomes (Fig. 3b). Similarly, transmigration and 3-D invasion of IFDUC1 MEC was stimulated, demonstrating that the normal breast ADSC secretome can influence cancer cells (Fig. 3c).

### 3-D invasion of NORMA MEC and IFDUC1 MEC is specifically stimulated by IL-6 and IL-8

Since ADSC secretomes enhanced invasion of NORMA and IFDUC1 MEC, compared to the control (Fig. 3d) we tested if IL-6 as well as IL-8 could stimulate invasion of NORMA MEC and IFDUC1 (Fig. 3d). IL-6 significantly stimulated invasion of all four NORMA cell lines. When NORMA2 MEC was incubated with IL-8 invasion was significantly increased compared to the control. IFDUC1 MEC showed the highest inherent invasion potential compared to all NORMA MEC. Microscopically, morphological changes of epithelial MEC into mesenchymal and amoeboid cell types were observed when invading a 3D collagen matrix (Fig. 3e).

### A direct co-culture of NORMA MEC and ADSC significantly stimulates the proliferation of MEC

**Figure 4:**
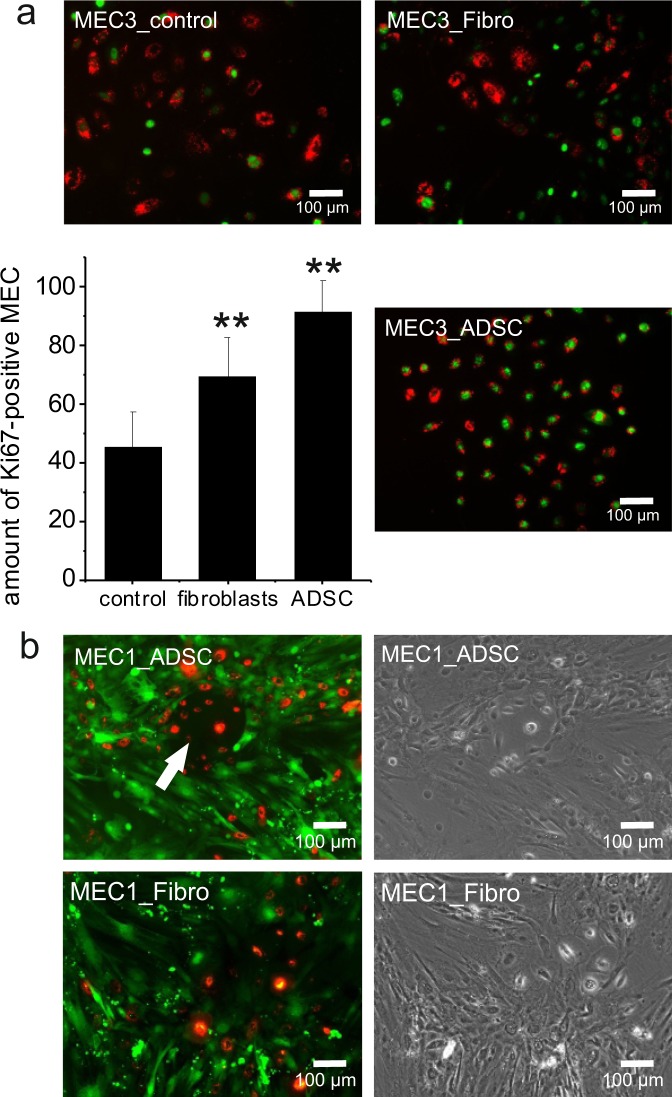
Direct co-cultures of ADSC and NORMA MEC. Direct co-cultures of ADSC with NORMA1-5 MEC highly significantly stimulated the Ki67 marker for proliferation (y-axis in percent ± SD) of NORMA MEC when compared with co-cultures of fibroblasts and MEC control monocultures (**p ≤ 0.01). Double-labeled NORMA3 MEC [red fluorescent dye (CM-DiI) and immunofluorescence staining of Ki67 (green)] were counted and represented as percentages of Ki67-positive cells. As an example, double-labeled NORMA3 MEC, CM-DiI-labeled and Ki67-positive cells, are shown (**a**). For further microscopic evaluation and as an example, ADSC1 were stained with a green fluorescent cell membrane dye (Oregon green) and NORMA1 MEC with a red fluorescent dye (CM-DiI). Note cellular interactions of ADSC1 with NORMA1 MEC forming colonies (white arrow), however, these interactions were not observed in the control group with fibroblasts (**b**).

In direct co-culture experiments with ADSC, a significantly higher amount of expression of the proliferation marker Ki67 used in immunocytochemistry was noted for all patient derived NORMA1-5 MEC compared to direct co-culture with fibroblasts or NORMA MEC monocultures (Fig. 4a). NORMA MEC expressed Ki67 significantly more with ADSC direct co-cultures compared to monocultures. Additionally, in direct co-cultures, NORMA MEC formed small colonies in many areas which were surrounded by ADSC (Fig. 4b, arrow). At the colony borders, cell-cell interactions were apparent. In contrast, in direct ADSC co-cultures with fibroblasts we observed virtually no colony forming with NORMA MEC (Fig. 4b).

### The ADSC secretome induces changes of gene expression of NORMA MEC

To test for specific changes in gene expression, we evaluated the influence of the ADSC secrotome on NORMA MEC using indirect co-cultures.

**Figure 5:**
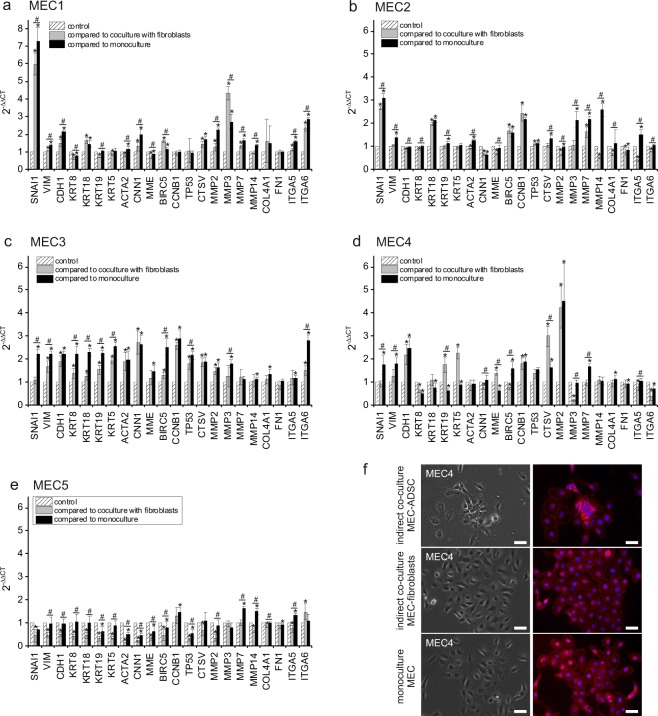
Gene expression of NORMA1-5 MEC in indirect co-cultures with ADSC. Relative expression in 2^−ΔΔCT^ (y-axis) ± SEM representing different genes (x-axis) of NORMA1-5 MEC in ADSC indirect co-cultures of individual patients compared to indirect co-culture with fibroblasts and monoculture. Grey bars indicate the relative expression of NORMA1-5 MEC in indirect (indir.) co-cultures with ADSC compared to indirect co-cultures with fibroblasts, black bars indicate the relative expression of NORMA1-5 MEC in indirect co-cultures with ADSC compared to monocultures. Controls were set to 1 (hatched bars) (**a–e**). An example showing membrane staining of NORMA4 MEC is shown in (**f**). (*/#p ≤ 0.05) (bar = 50 µm).

For example, NORMA3 MEC demonstrated a significant 1-3-fold increase in genes such as *SNAI1, vimentin (VIM), KRTs, alpha smooth muscle actin (ACTA2), MME/CD10, calponin (CNN1), BIRC5, CCNB1, TP53, CTSV* compared to monocultures and in part compared to indirect co-culture with fibroblasts (excl. *SNAI1* and *MME/CD10*) (Fig. 5c). The effect on gene expression was significantly higher for most genes in the ADSC co-cultured group compared to monocultures than compared to the indirect co-culture with fibroblasts (Fig. 5a–e). NORMA4 MEC expression of *SNAI1, VIM, E-cadherin (CDH1), BIRC5, CCNB1, TP53*, and *CTSV* was also significantly increased with ADSC4 co-culture while there was a significantly lower expression of the *KRTs, MME/CD10* compared to monocultures (Fig. 5d). Compared to the indirect co-culture with fibroblasts, there was no change in the expression of some genes, such as *SNAI1, VIM* and *KRT18*. However, we detected a significant increase in *CDH1, KRT19, KRT5, MME/CD10, CCNB1, TP53, CTSV, MMP2*, supporting an influence of differentiated fibroblasts (Fig. 5d). Inherent MMP expression of NORMA MEC for comparison. was relatively low supporting a non-invasive phenotype (Supplemental Fig. 1). Interestingly, after cultivation in ADSC co-cultures, expression of MMPs increased significantly for most NORMA MEC, e.g. *MMP2*, *MMP7, MMP14* in four NORMA MEC and *MMP3* in three NORMA MEC supporting a further influence of the ADSC secretome on invasion mediated by MMP activation and extracellular matrix (ECM) digestion.

Although expression of ECM proteins such as *COL4A1* or *FN1* was not significantly influenced by the ADSC secretome, receptors for ECM proteins e.g. *ITGA5* were significantly induced in all NORMA MEC and *ITGA6* in three NORMA MEC compared to monocultures. This demonstrates an ADSC secretome effect on ECM receptor induction. Lastly, microscopically, for all groups, NORMA MEC showed typical colony formation (Fig. 5f).

## Discussion

Reconstructive breast surgery using autologous lipotransfer as a replacement filling material has become a widely used procedure. This present study focused on normal breast cells and brings forth new knowledge regarding the biology of normal breast cell and ECM interactions. For example, we demonstrate a clear dependence of NORMA MEC on the ADSC secretome, which positively influences functions including proliferation, migration, invasion along with distinct changes in gene expression, like upregulation of MMPs and integrin receptors specific for ECM regulation. The ADSC secretome similarly influenced the same functions of primary breast cancer cells. We support the idea that understanding normal breast cell interactions is necessary in order to translate these findings into more complex cell behaviors in breast cancer.

Many clinical studies claim there is no effect on breast cancer recurrence following lipotransfer compared to patients without lipotransfer (reviewed in^19,20,22^). On the other hand, there are two recently published clinical studies in which subgroups of patients had a higher risk for local tumor recurrence^28,29^. Concerns have been noted due to possible biases in the matched population^30^. Such contradictory results could be explained by small patient groups, short follow-up periods missing or inadequate control groups, retrospective analyses, lack of standardization of surgical techniques and harvesting methods^22,31^. In the case of partial mastectomy, thus breast conserving surgery, a possible effect of ADSC on both normal MEC and residual mammary epithelial cancer cells is likely.

Previous studies have shown that ADSC stimulate functional properties of breast cancer cells^16–18^. The influence of ADSC on normal MEC was investigated in a few studies, where in part a positive effect on proliferation was shown^32–34^. In a another study normal MEC proliferation was also enhanced along with Heparanase/MMP-9 gene expression by ADSC and adipocytes^35^. We noted in this present study that NORMA MEC and IFDUC1 MEC viability and proliferation were significantly stimulated by the ADSC secretome and in direct co-cultures with ADSC. Further, proliferation was also enhanced in direct co-cultures with fibroblasts. This could be due to various proteins that are secreted by dermal fibroblasts^36^ which can induce cell proliferation. At the gene expression level we detected a significantly higher expression of *BIRC5/Survivin* and *CCNB1* of NORMA2 MEC and NORMA3 MEC with ADSC secretomes compared to controls. *Survivin* is an inhibitor of apoptosis while *CCNB1* is a key initiator in mitosis. *Survivin* expression is found in healthy breast tissue and benign breast tumors, probably due to proliferative or “dysplastic luminal epithelial cells”^37^, and plays a role in tissue remodeling. High levels of both *CCNB1* and *Survivin* have also been noted in breast cancer^38,39^. In contrast to our findings, in a study from Duss *et al*. proliferation of primary MEC with ADSC was reduced in co-cultures where many genes – e.g. cell cycle genes – were downregulated^32^ but this could also be due to hypoxic conditions (5% O_2_)^32^. Taken together, the ADSC secretome positively influences MEC to proliferate and this could regenerate mammary tissue or even lead to a transition of MEC into other normal cell phenotypes. On the other hand it may also influence proliferation of residual breast cancer cells following resection and lipotransfer.

We previously noted that both NORMA and IFDUC1 ADSC secretomes isolated from consecutive stages of adipocyte differentiation significantly stimulated induction of the epithelial to mesenchymal (EMT) and the epithelial to amoeboid transition (EAT) leading to invasion of IFDUC1 MEC^23^. IFDUC1 mesenchymal (MES) cells showed high intrinsic levels of invasion but no further induction was noted following the same treatment^23^. When compared to four different carcinoma ATCC cell lines, IFDUC1 MES demonstrated the highest invasiveness and migrated at the highest velocity through 3D channels^40^. In this present report we now demonstrate that ADSC secretomes isolated from non-differentiating ADSCs (normal breast) also significantly stimulate migration and invasion of IFDUC1 MEC similarly to NORMA MEC.

In concordance with a migration and invasion phenotype, gene expression results of NORMA MEC cells cultured with ADSC secretomes using co-culture conditions resulted in induction of EMT markers. For example, NORMA2, 3 and 4 MEC grown with ADSC secretomes induced the typical EMT marker genes *SNAI1* and *VIM*. The EMT is a well-known process found during wound healing and tissue regeneration in case of injury and chronic inflammation^41^ where migration capacities are essential. However, it has also been reported to increase the migratory and invasive capacities of breast cancer cells.

Significantly higher expressions of *CDH1/E-cadherin, KRT19/5, MME/CD10, CTSV/cathepsin V* or *cathepsin L2*, and *MMP2, 3, 7, 14* were found in NORMA MEC co-cultures with ADSC compared to fibroblasts. MMPs are zinc-dependent endopeptidases with the main function to digest the ECM. In breast tissue, MMPs function to facilitate ductal expansion through the basement membrane and promote branching morphologies due to interaction with cytokines, growth factors and TNFs, which were high in our ADSC secretomes^42^. MMPs have been implicated in cancer invasion and metastasis. In breast cancer, normal MMP remodeling functions become aberrant leading to tumor progression and metastasis^42^. *MMP-2* degrades collagens. Normal MEC *MMP-2* is generally expressed to a low extent^43^ and in the present study was virtually not detectable in intrinsic NORMA1-5 MEC (Supplemental Fig. 1). *MMP-2* has also been described as an indicator of normal tissue remodeling^44^, but is higher expressed in tumors with a high invasion potential^45^. *CTSV* codes for a lysosomal cysteine proteinase, known as a breast-cancer related gene^46^ and potentially involved in the tumor process^47^. We support the idea that the ADSC secretome induced key genes which positively influenced migration and invasion.

A distinct influence of ADSC on NORMA MEC phenotypes such as basal, luminal, myoepithelial cells by gene expression analyses was also observed in our investigation. The simultaneous growth of different MEC phenotypes is mandatory for the formation of a hierarchical architecture normally found in breast tissue. For example, the ADSC secretome induced expression of the basal/myoepithelial markers *KRT5, ACTA2, CNN1/calponin* in NORMA2 MEC and luminal-specific *KRT8, 18* and *19* were significantly higher expressed in NORMA4 MEC compared to the control. Additionally, *CDH1* and *MME/CD10* were significantly increased, indicating the presence of a high amount of progenitor cells^48^. Similarly, mammosphere assays and flow cytometry demonstrated that ADSC co-cultures with both normal MEC and breast cancer cell lines, or the treatment with ADSC secretome altered the number of luminal progenitor cells^34^. Since these cell types have a higher capacity to transform, the authors concluded that this could lead to an increasing susceptibility to develop breast cancer^34^. Lastly, myoepithelial markers like *CNN1/calponin* and *ACTA2* were also significantly higher compared to control. Thus, we conclude that the ADSC secretome promotes luminal and basal phenotypes with enrichment of progenitor cells but also myoepithelial markers of NORMA MEC.

We conclude that the influence of the ADSC secretome on the functions NORMA MEC and IFDUC1 MEC is due to key proteins. In line with our study, Kauts *et al*. showed that CCL5/RANTES was highly expressed in ADSC^49^. CCL5 is an inflammatory chemokine known to mediate cross-talk between tumor cells and the tumor environment resulting in increasing amounts of tumor cell migration and invasion^50^. ADSC are a well-known source of CXCL12 (SDF1-alpha)^16^. CXCL12 is a chemokine and via the CXCR4/CXCL12 axis of breast cancer cells induces a chemotactic response and invasion^51^.

All ADSC patient secretomes also demonstrated high protein levels of IL-6, IL-8, CXCL1 and MCP-1. CXCL1 is a chemotactic chemokine and is expressed and secreted from breast cancer stromal cells and has been correlated with poor patient prognosis, high tumor growth and metastasis^52^. Yoshimura *et al*. could show that breast tumor cells and stromal cells produced the chemokine MCP-1^53^. MCP-1 has been associated with macrophage infiltration, angiogenetic activity, and tumor growth and migration^54,55^.

In line with our findings, it was previously shown that IL-6 and IL-8 ADSC secretion stimulates EMT, migration and invasion of breast cancer cell lines and enhances the malignant properties of colon cancer cell lines^16,18^. Further, proliferation of the normal MEC cell line MCF-10A was slightly increased in ADSC CM containing these interleukins^16^. From the literature it is known that adipose tissue (visceral fat) is an important source for IL-6 secretion and most probably plays a role in systemic inflammation of obese people^56^. This chronic inflammation could contribute to tumor progression and obesity-related cancer incidence^18^. It is also known that patient’s IL-6 and IL-8 serum levels can correlate with breast cancer disease stage^57^ and treatment of patients with IL-6 antibodies is currently under investigation (reviewed in^58^). Further, Sansone *et al*. concluded that mammospheres from normal gland can attain malignant properties after incubation with IL-6^59^.

The concentration of ECM proteins in the ADSC secretome was nearly equal in all 5 patients, e.g. FN/fibronectin and THBS1/thrombospondin-1. ADSC secretion of several types of ECM proteins^60^, supports adhesion and proliferation of MEC. Although, the ADSC secretome did not influence ECM *COL4A1* and *FN1* gene expression of NORMA MEC, the integrin receptors *ITGA5/6* were induced. This supports ADSC mediated MEC ECM interactions are essential for development. Normal mammary breast tissue contains low levels of FN, compared to higher levels in breast tumors^61^. Recently, it could be shown that high FN concentration in breast tumors induce EMT and might be both, a cause and result of tumor initiation and/or progression^61^. In contrast, THBS1 is an ECM glycoprotein that inhibits tumor cell growth and metastasis partly due to its anti-angiogenic effect^62^. However, prolonged delivery of THBS1 can result in emerging resistant cells that lead to tumor insensitivity^62^.

HGF could be found in all ADSC secretome from all patients, but at very low amounts in ADSC2. HGF is a potent mitogen for a range of different cell types, especially endothelial and epithelial cells. An additional potent angiogenic factor detected in the ADSC secretome is bFGF (measured in all ADSC CM, except ADSC5 CM). Both cytokines can induce angiogenesis and therefore can be regarded as supporting cytokines for the remodeling and the integration of the lipotransplant in the defect area.

ENPP2/autotaxin (ATX) was another enzyme present in the ADSC secretome. ATX converts lysophosphatidylcholine (LPC) into lysophosphatidic acid (LPA) that itself has diverse effects on various cells. Many tumors are known to secrete ATX. We recently showed that ADSCs and adipocytes are the main cellular source of ATX in both normal and breast tumor tissues^63^. Benesch *et al*. demonstrated ATX secretion in adjacent adipose tissue of breast tumors^64^. The increased LPA concentration increased the inflammatory cytokine secretion in tumors leading to a vicious cycle towards a higher LPA secretion etc.^64^.

## Conclusions

We demonstrate in this study that patient-matched ADSC secretomes significantly stimulated normal MEC and IFDUC1 MEC functional properties including proliferation, migration and invasion and influenced gene expression patterns. Our findings support key cellular interactions of normal breast biology. With regard to the usage of ADSC for breast reconstruction this could be beneficial, because of the local stimulation of tissue-resident normal MEC. By secretion of a range of cytokines, ADSC can induce proliferation and migration and remodeling of NORMA MEC and further recruit other cells to support the integration of the lipotransplant. However, even with a small sample size of n = 5, we could conclude that ADSC from different patients exhibit different capabilities in their influence upon breast cells. Therefore, the decision of using lipotransfer, whether cell-assisted or not, should carefully be chosen and possible risks should be discussed with patients. We propose our findings could translate to residual breast cancer cells not cleared from the immune system. One future recommendation could be that candidates for a lipotransfer would represent low risk breast cancer patients with early stage disease and with a lower risk for recurrence or metastasis than patients with later stages.

## Supplementary information

Supplemental Figure 1

## Data Availability

The datasets generated during and/or analysed during the current study are available from the corresponding author on reasonable request.
