# Supplementary material for: Plasticity of patient-matched normal mammary epithelial cells is dependent on autologous adipose-derived stem cells
Source: Sci Rep. 2019 Jul 24;9:10722. doi: 10.1038/s41598-019-47224-2 (PMC6656715; doi:10.1038/s41598-019-47224-2)
Supplement: Supplementary file 1 — Supplemental Figure 1 [file 41598_2019_47224_MOESM1_ESM.docx]

**Plasticity of patient-matched normal mammary epithelial cells is dependent on autologous adipose-derived stem cells**

Annika Kengelbach-Weigand*^,1^, Kereshmeh Tasbihi^1^, Pamela L. Strissel², Rafael Schmid^1^, Jasmin Monteiro Marques^1^, Justus P. Beier^1,3^, Matthias W. Beckmann², Reiner Strick², Raymund E. Horch^1^, Anja M. Boos^1^

1 Department of Plastic and Hand Surgery and Laboratory for Tissue Engineering and Regenerative Medicine, University Hospital of Erlangen, Friedrich-Alexander University of Erlangen-Nürnberg (FAU), Erlangen, Germany, phone +49 9131-85-33277, fax +49 9131-85-39327

2 Department of Obstetrics and Gynecology, University Hospital of Erlangen, Laboratory for Molecular Medicine and Comprehensive Cancer Center Erlangen-EMN (CCC), Friedrich-Alexander University of Erlangen-Nürnberg (FAU), Erlangen, Germany, phone +49 9131-85-33553, fax +49 9131-33456

3 current affiliations: Department of Plastic Surgery, Hand and Burn Surgery, University Hospital RWTH Aachen, phone +49 241 80-89700, fax +49 241 80-82448

**Supplemental Fig. 1**

**MMP expression of NORMA MEC lines**

Bar graphs show a comparison (x-axis) between NORMA1-5 MEC of the relative expression (y-axis) of different genes. Gene expression results are presented as 2^-ΔCT^.
